# Human amniotic epithelial cells can differentiate into granulosa cells and restore folliculogenesis in a mouse model of chemotherapy-induced premature ovarian failure

**DOI:** 10.1186/scrt335

**Published:** 2013-10-14

**Authors:** Fangyuan Wang, Li Wang, Xiaofen Yao, Dongmei Lai, Lihe Guo

**Affiliations:** 1 The Center of Research Laboratory, The International Peace Maternity and Child Health Hospital, School of Medicine, Shanghai Jiaotong University, Shanghai 200030, China; 2 The IVF center, The International Peace Maternity and Child Health Hospital, School of medicine, Shanghai Jiaotong University, Shanghai 200030, China

## Abstract

**Introduction:**

Ovarian dysfunction frequently occurs in female cancer patients after chemotherapy, but human amniotic epithelial cells (hAECs) that can differentiate into cell types that arise from all three germ layers may offer promise for restoration of such dysfunction. Previous studies confirmed that hAECs could differentiate into cells that express germ cell-specific markers, but at this time hAECs have not been shown to restore ovarian function.

**Methods:**

To model premature ovarian failure, hAECs infected with lenti-virus carrying green fluorescent protein were injected into the tail vein of mice sterilized with cyclophosphamide and busulphan. hAECs migrated to the mouse ovaries and overall ovarian function was measured using immunohistochemical techniques.

**Results:**

Seven days to two months after hAECs transplantation, ovarian cells were morphologically restored in sterilized mice. Hemotoxylin and eosin staining revealed that restored ovarian cells developed follicles at all stages. No follicles were observed in control mice at the same time period. Immunostaining with anti-human antigen antibodies and pre-transplantation labeling with green fluorescent protein (GFP) revealed that the grafted hAECs survived and migrated to mouse ovary, differentiating into granulosa cells. Furthermore, the ovarian function marker, anti-Müllerian hormone, was evident in treated mouse ovaries after hAEC transplantation.

**Conclusions:**

Intravenously injected hAECs reached the ovaries of chemotherapy-treated mice and restored folliculogenesis, data which suggest promise for hAECs for promoting reproductive health and improving the quality of life for female cancer survivors.

## Introduction

Ovarian dysfunction or failure is common in premenopausal women receiving chemotherapy and risk of such can be predicted by the patient age and chemotherapeutic dosage. Approximately 30% of women under the age of 35, 50% of women aged 35 to 40 years, and 75 to 90% of women in their 40s experience permanent cessation of menstrual function after chemotherapy, leading to premature menopause [1]. *In vitro* studies reveal that chemotherapy induces apoptosis in pre-granulosa cells of primordial follicles, reducing the likelihood of successful ovulation [2]. As survival rates for young cancer patients continue to improve, protection against iatrogenic infertility caused by chemotherapy will be of greater priority [3,4].

Recently, a gonadotropin-releasing hormone, (GnRH) agonist has been used to minimize and prevent the gonadotoxic effect of chemotherapy in humans, and data show that patients treated with both a GnRH agonist and chemotherapy resumed spontaneous ovulation and menses or were able to conceive [5,6]. However, another stratagem for this therapy may be treatment of ovarian failure caused by chemotherapy.

Studies indicate that reproductive dysfunction can be treated by bone marrow stem cells. In 2005, Johnson and colleagues reported that bone marrow transplantation restored oocyte production in wild-type mice sterilized by chemotherapy, as well as in ataxia telangiectasia-mutated gene-deficient mice, which are otherwise incapable of making oocytes [7]. In 2007, the same group reported that bone marrow transplantation generated immature oocytes and rescued long-term fertility in a mouse model of chemotherapy-induced premature ovarian failure [8]. However, Eggan and colleagues reported that the circulating bone marrow cells could not generate ovulated oocytes. Instead, cells that travelled to the ovary through circulation had characteristics of committed blood leukocytes [9]. Recently, Santiquet and co-workers reported that there was no evidence that transplanted bone marrow cells provided new fertilizable oocytes in a mouse model treated with chemotherapeutic agents or with bovine embryonic ovarian tissue grafts. However, transplanted bone marrow cells did improve the fertility of severe combined immunodeficiency (SCID) mice previously treated with chemotherapeutic agents [10].

Human amnion epithelial cells (hAECs) derived from term placentas are anatomically and histologically specialized fetal epithelial cells and may maintain the pluripotent properties of early epiblast cells. hAECs have been shown to have the potential to differentiate *in vitro* into three embryonic germ layers [11-13]. hAECs are discarded post-partum and can be expanded extensively in culture, so they may offer an alternate source useful for regenerative medicine and cell therapy [14].

The formation of germ cells from the epiblast in the embryo during gastrulation involves segregation of the primordial germ cells (PGCs) from their somatic lineages and migration of PGCs to the genital ridges. Because hAECs develop from epiblasts prior to gastrulation, hAECs may have the potential to differentiate into germ cells [15]. Recently, Evron reported that hAECs cultured in media containing serum substitute supplement (SSS) can differentiate into oocyte-like cells and express germ cell specific markers [16].

Here, hAECs have been used to clarify the effects of cell transplantation on female reproductive function using a preclinical mouse model of chemotherapy-induced premature ovarian failure. In this study we show - for the first time - that hAECs can be grafted into the ovaries of chemotherapy-treated mice and restore ovarian function.

## Material and methods

### Preparation and culture of hAECs

Human placentas were obtained at term pregnancy during uncomplicated Caesarean sections with written and informed consent from woman who tested negative for HIV-I, and hepatitis B and C. The indication for Caesarean section is breech presentation, repeat operation, fetal distress and twins. The institutional ethics committee approved the use of human amnions for this project. The amniotic membranes were mechanically peeled from the chorionic portion of the placenta placed in 250-ml flasks containing RPMI 1640 medium, and cut with a razor to yield 0.5 to 1.0 cm^2^ segments. Placental segments were digested with 0.25% trypsin/EDTA at 37°C for 45 minutes. The resulting cell suspensions were seeded in a six-well plate in RPMI 1640 medium supplemented with 10% FBS (PAA Laboratories GmbH, Cölbe, Germany), streptomycin (100 U/mL; Gibco, Grand Island, NY, USA), penicillin (100 U/Ml; Gibco, Grand Island, NY), and glutamine (0.3 mg/Ml; Gibco), and incubated at 37°C 5% CO_2_ in humidified air. Once hAECs reached 80 to 90% confluence, cells were ready for experiments.

### RNA extraction and real-time qPCR analysis

Total RNAs were isolated from samples using an RNeasy Mini Kit (Qiagen, Chatsworth, CA, USA). Five hundred nanograms of total RNA from each sample were used in reverse transcription (RT) using an iScript cDNA synthesis kit (Bio-Rad, Hercules, CA, USA). Real-time RT-qPCR was performed on cDNA using IQ SYBR Green (Bio-Rad) on the Mastercycler® ep realplex (Hamburg, Germany). All reactions were performed in a 25-μl volume. Primer sequences are listed in Additional file 1: Table S1. Reaction conditions for *STELLA*, *STRA8* and *DAZL* were: 94°C for 2 minutes, then 94°C for 30 sec, 60°C for 30 sec, 72°C for 45 sec, 28 cycles, then 72°C for 10 minutes; conditions for *OCT4*, *CD117*, *HLA-DR*, *BLIMP1*, *VASA*, *SCP1*,*SCP3* and *18 s RNA* were: 94°C for 2 minutes, then 94°C for 30 sec, 53°C for 30 sec, 72°C for 45 sec, 28 cycles, then 72°C for 10 minutes; conditions for *NANOG* and *c-MOS* were: 94°C for 2 minutes, then 94°C for 30 sec, 60°C for 30 sec, 72°C for 45 sec, 28 cycles, then 72°C for 10 minutes.

### Animals

C57BL/6 wild-type female mice, six-weeks-old (18.15 ± 0.16 g by weight), were sterilized by intraperitoneal injection of busulfan (Sigma-Aldrich, 30 mg/kg; resuspended in dimethyl sulfoxide (DMSO)) and cyclophosphamide (Sigma-Aldirich, 120 mg/kg; resuspended in DMSO) were used as recipients [17]. Normal control mice were injected with DMSO only. All procedures for animals were approved by the Institutional Animal Care and Use Committee of Shanghai, and were performed in accordance with the National Research Council Guide for Care and Use of Laboratory Animals.

### Transfection and transplantation of hAECs

The hAECs grown to a density of 80 to 90% were used, and lenti-virus enhanced with green fluorescent protein (EGFP, a gift from Tianjin Liu) [18] was added to cultured cells and incubated for 24 h. Titration of concentrated supernatants was performed by serial dilutions of vector stocks on 1 × 10^5^ Hela cells followed by fluorescence-activated cytometric analysis according to the formula: 1 × 10^5^ Hela cells × % EGFP positive cells × 1,000/μl virus. Titers of lenti-viral vectors were 1× 10^8^ - 1 × 10^9^ TU/ml. After growing for another two days, hAECs were examined by fluorescence microscopy. The overall cell transfection rate was determined to be greater than 95%. After lenti-viral infection for two days, hAECs were washed three times and trypsinized (0.25% trypsin), neutralized in 10% FBS, washed with phosphate-buffered saline (PBS) and resuspended in the culture medium.

To transplant hAECs into mouse ovaries, mice were anesthetized with an intraperitoneal injection of pentobarbital sodium (45 mg/kg ). Approximately 6 μl of a single-cell suspension (2 × 10^6^ cells), or 6 μl of culture medium for the untreated control group, were injected into mouse tail veins one week after chemotherapy.

### Immunohistochemical analysis

Ovaries from treated and control animals were fixed with 4% paraformaldehyde (4°C, overnight), dehydrated through a graded ethanol series, vitrified in xylene, and embedded in paraffin. Sections (6-μm thick) were fixed for 5 minutes in neutral buffered formalin, after which endogenous peroxidase activity was quenched by incubating the sections in 0.3% hydrogen peroxide in methanol for 30 minutes. Sections were treated with mouse anti-human anti-Müllerian hormone (AMH, 1:30; AbD Serotec, Oxford, United Kingdom), anti-human specific nuclear antigen (monoclonal antibody, 1:300; EMD Millipore, Darmstadt, Germany), or mouse anti-human follicular stimulating hormone receptor (FSHR, 1:100; Abcam, Cambridge, England) for antibody detection. Peroxidase reaction kits (Vector Laboratories, Burlingame, CA, USA) were used according to the manufacturer’s instructions. Peroxidase substrate was developed by using a DAB (3,39-diaminobenzidine) substrate kit (Vector Laboratories). Slides were counterstained with hematoxylin QS (Vector Laboratories) and were either mounted with low viscosity aqueous mounting medium (Scytek Laboratories, Logan, UT, USA) or dehydrated and mounted with VectaMount Permanent Mounting Medium (Vector Laboratories).

### Immunofluorescence staining

hAECs were fixed with 4% paraformaldehyde for 15 to 20 minutes at room temperature, and then washed twice (10 minutes each) with 1 × PBS. Cells were permeabilized with 0.1% Triton X-100 for 10 minutes at room temperature, and then washed twice with 1 × PBS. Then cells were blocked with blocking solution for 30 minutes and incubated with anti-OCT4 (rabbit anti-human 1:200, Santa Cruz Biotechnology, Santa Cruz, CA, USA), anti-VASA antibody (goat antihuman 1:200, Santa Cruz), anti-DAZL (goat anti human 1:500, Santa Cruz), anti-STELLA (goat anti Human 1:200, Santa Cruz), anti-NANOG (rabbit anti human 1:200, Millipore), anti-CD117 (Rabbit antihuman 1:800, eBioscience, CA, USA) antibody for 1 h at room temperature. Ovaries from treated and control animals were fixed with an optimal cutting temperature (OCT) compound (Sakura Finetek, Seattle, USA) and 5-μm thick fresh sections were made. Slides were washed twice with PBS and blocked with blocking solution for 30 minutes at room temperature and then incubated overnight at 4°C with rabbit polyclonal anti-GFP (dilution 1:200; Chemicon, Massachusetts, USA) or mouse anti-human nuclear monoclonal antibody (dilution 1:50; Millipore, Massachusetts, USA). Then cells or sections were washed three times with 1 × PBS, and probed with FITC-labeled IgG (1:200, Santa Cruz, CA, USA) or Rodamine (TRITC)-labeled IgG (1:100, Invitrogen, CA, USA). Fluorescence images were obtained with a Leica DMI3000 microscope (Heidelberg, Germany).

### Statistics

Means for relative gene expression were compared by ANOVA using Microsoft Excel software.

## Results

### hAECs express stem cell markers, but not germ cell markers

**Figure 1:**
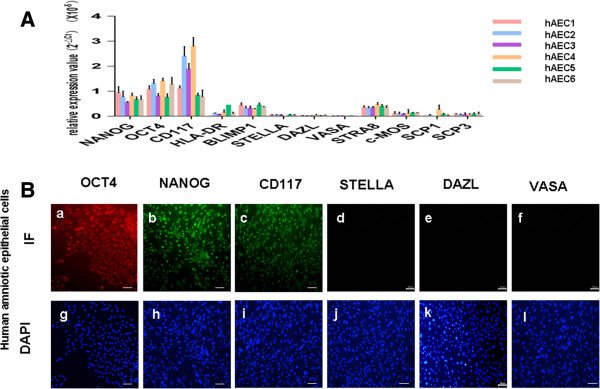
**Human amniotic epithelial cells (hAECs) did not express germ-cell-specific genes. (A)** Quantitative PCR was used to analyze germ-cell-specific expression in hAECs. CT values were expressed as a percentage of 18S RNA (18S = 100%) and used to calculate mean normalized expression relative to 18S. Results are shown as mean and standard deviation of three experiments. **(B)** Immunofluorescence analysis of germ-cell-specific genes in hAECs. Note that hAECs expressed OCT4 (red), NANOG (green) and CD117 (green), but did not express DAZL, STELLA or VASA. DAPI staining for nuclei. Scale bars = 50 μm.

To examine germ cell-specific genes in hAECs, six independent hAECs were cultured for one week. A portion of the attached cells were collected and assayed. Real-time PCR was used to assess markers expressed by hAECs (See Additional file 1: Table S2). As expected from previous reports [11,12], we observed consistent expression of the human *OCT4* and *NANOG* gene in all six samples. The cells also expressed markers of the surface antigen *C-KIT*(*CD117*); however, major histocompatibility (MHC) Class II (*HLA-DR*) expression was modestly expressed (Figure 1A). Next we measured expression of germ-cell-specific genes in hAECs, including *BLIMP1* (B-lymphocyte-induced maturation protein 1), *STELLA* (developmental pluripotency-associated protein 3, stella-related protein), *DAZL* (deleted in azo-ospermia-like), *VASA* (probable ATP-dependent RNA helicase DDX4, vasa homolog), *STRA8* (stimulated by retinoic acid gene 8), *c-MOS* (oocyte maturation factor mos), and *SCP1* and *SCP3* (syntaptonemal Complex Protein 1 and 3). Comparatively, mRNA expression of pre-meiotic germ genes, including *BLIMP1* and *STRA8,* were low, and *STELLA*, *DAZL*, *VASA* and the meiotic genes, such as *SCP1* and *SCP3*, were lower still (Figure 1A). Consistent with the transcriptional profiles, OCT4, NANOG and CD117, expressions were detectable in hAECs at the protein level using immunofluoresence. However, DAZL, STELLA and VASA protein were not detectable in these cells (Figure 1B). Consistent with a previous study [16], these data suggest that germ cell markers are not expressed in hAECs.

### hAECs rescues oocyte production in chemically-damaged ovaries

**Figure 2:**
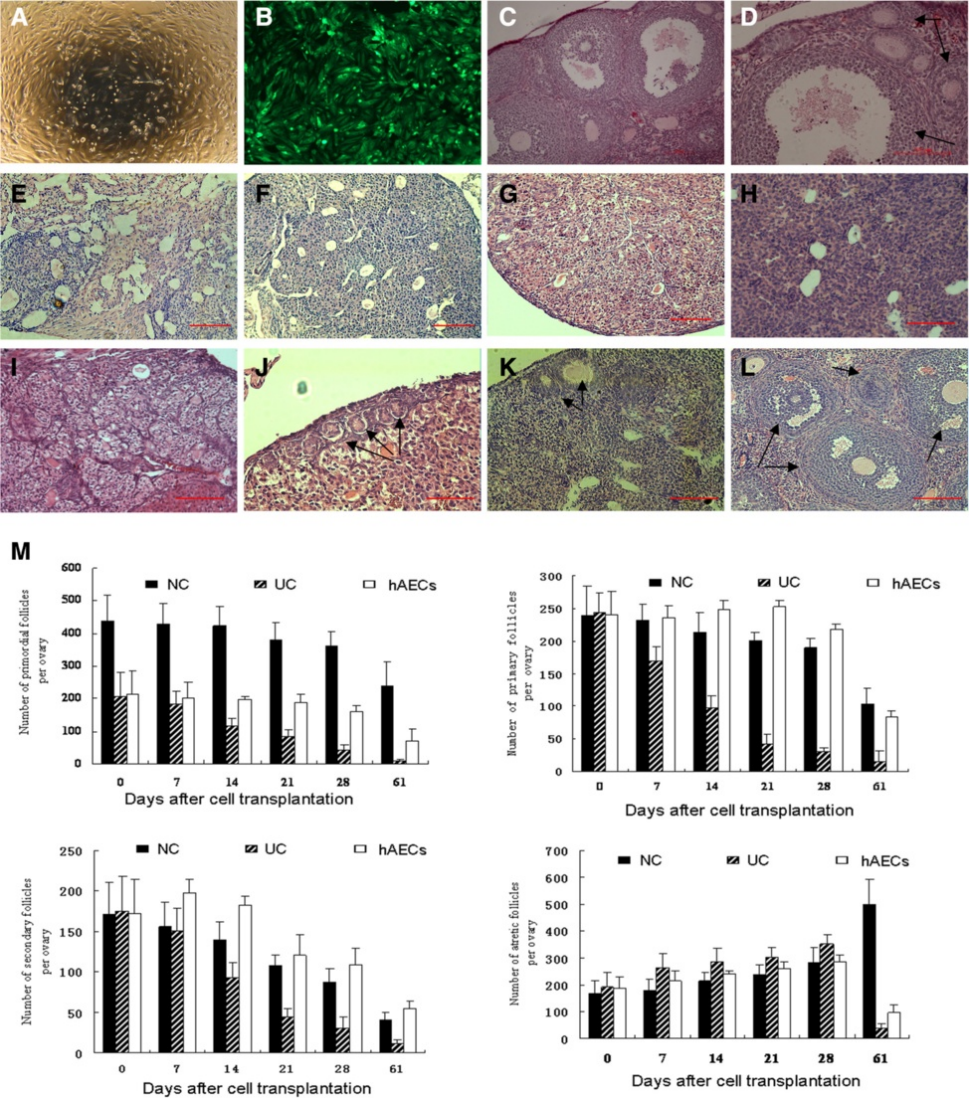
**Transplantation of GFP-transfected hAECs into sterilized recipient mice and the follicle activation induced by hAECs transplantation. (A)** hAECs cells grown to 85% density. **(B)** GFP-transfected hAECs. Representative H&E micrographs of ovary sections from: non-sterilized normal control mice **(C**, **D)**; sterilized non-transplanted mice after a 7-day recovery period **(E)**, a 14-day recovery period **(F)**, a 21-day recovery period **(G)** and a 2-month recovery period **(H)** showing stroma, and atretic primordial or primary follicles; sterilized recipient mice 7 days **(I)**, 14 days **(J)**, 21 days **(K)** and 2 months **(L)** after transplantation of hAECs. No obvious follicles were observed in recipients seven days after hAEC transplantation **(I)**; however, the hollow structure destroyed by chemotherapy was reduced and obviously compared with **(E)**. Primordial follicles are visible in J, primary follicles were visible in K and large antral follicles are shown in L. Arrows indicated follicles at various stages of maturational development. Scale bars = 100 μm. **(M)**. Differential follicle counts of primordial, primary, secondary and atretic follicles in ovaries of each groups. NC, Normal control; UC, Untreated control; hAECs, hAECs transplantation group. GFP, Green fluorescent protein; hAECs, Human amniotic epithelial cells.

To assess whether hAECs could migrate to ovaries and restore ovarian function, wild-type female mice were sterilized by pre-treatment with cyclophosphamide and busulfan to destroy the existing pre- and post-meiotic germ cell pools [19,20]. These mice were used as “hAECs recipients”. The hAECs grown to 85% density were infected with lenti-virus carrying GFP (Figure 2A, B). After infection and culture for an additional week, 2 × 10^6^ hAECs were transplanted by tail vein injection into recipient females seven days after chemotherapy (n = 20). The untreated control group received 6 μl of culture medium (n = 15). No transplant-related deaths occurred.

**Figure 3:**
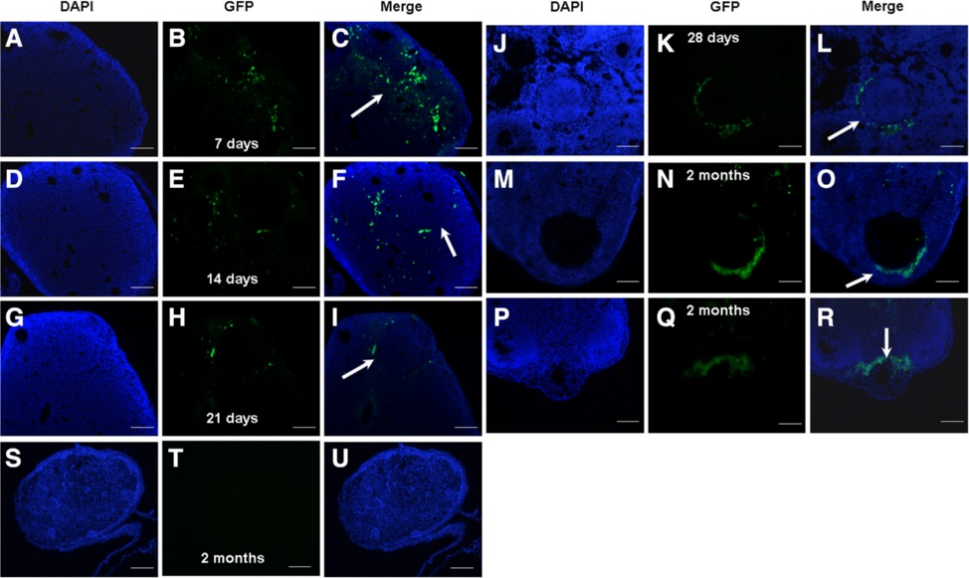
**Macroscopic appearance of recipient ovaries seven days to two months after transplantation with GFP-transfected hAECs. (A**-**F)** GFP staining dots shown in recipient ovaries 7 to 14 days after transplantation with GFP-transfected hAECs, whereas no GFP signal was observed in follicles. Follicles containing GFP-positive (green) cells were shown in recipient ovaries 21 days **(G**-**I)**, 28 days **(J**-**L)** and 2 months **(M**-**R)** after transplantation with GFP-transfected hAECs. **(S-U)** Oocytes in recipients without hAECs transplantation had no GFP signal after a two-month recovery period. Arrows indicate the GFP distribution pattern in ovaries. Blue, DAPI immunofluorescence. Scale bars, 200 μm **(S**-**U)**, 100 μm **(A**-**R)**. GFP, Green fluorescent protein; hAECs, Human amniotic epithelial cells.

As shown in Figure 2, seven days to two months after transplantation, mouse ovaries were collected, counted and assayed for the presence of oocytes as determined by their morphological appearance and expression of GFP. Histological evaluations seven days to two months after chemotherapy revealed that the chemotherapy regimen essentially destroyed total immature (primordial, primary, secondary) follicles in recipient ovaries which did not receive hAEC transplantation (Figure 2M). Untreated control ovaries contained little more than stromal and interstitial cells with an increasing atretic follicle and they lack GFP expression (Figure 2E-I, M, Figure 3S-U). However, GFP expression was observed and presented as distributed “found as dots” in these ovaries (Figure 3A-C). Ovaries of mice receiving hAECs transplantation at 14 days and 21 days possessed primary and developing follicles (Figure 2J, K, M), in which GFP expression and GFP-positive follicles were found (Figure 3D-I). By comparison, ovaries of mice receiving hAECs two months after chemotherapy possessed immature and mature oocyte-containing follicles (Figure 2L), including GFP-positive follicles (Figure 3J-R), similar to ovaries that had not been sterilized (Figure 2C, D, controls were injected with DMSO only, n = 10).

Interestingly, GFP-labeled cells were irregularly distributed seven days after hAEC transplantation (Figure 3A-C), and these were subsequently detected in ovarian tissue near the follicles 14 days (Figure 3D-F), 21 days (Figure 3G-I) and 28 days (Figure 3J-L) after hAECs transplantation, finally proximally situated near the follicles two months after hAECs transplantation (Figure 3M-R).

These experiments demonstrated that follicles can be regenerated in sterile females by hAECs transplantation.

### hAECs-derived cells infiltrate into the chemical-damaged murine ovarian tissue

**Figure 4:**
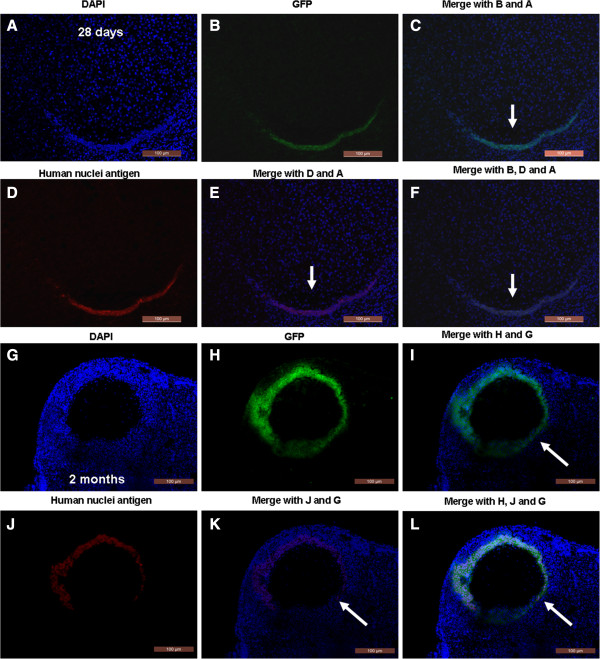
**Double-staining with GFP and human nuclear antigen to observe GFP-positive cells after hAECs transplantation. (A**-**F)** Ovarian sections stained with GFP and anti-human nuclear antibody reveal grafted hAECs 28 days after hAEC transplantation. **(G**-**L)** GFP staining co-localized with human nuclear antigen in antral follicles of recipient ovaries two months after hAEC transplantation. Arrows indicate a double-staining pattern in ovaries. Scale bars, 200 μm **(G**-**L)**, 100 μm **(A**-**F)**. GFP, Green fluorescent protein; hAECs, human amniotic epithelial cells.

To confirm whether GFP-positive cells in recipient ovaries were indeed derived from grafted hAECs, we performed double-staining with GFP and human specific nuclear antigen in recipient ovarian sections 28 days or 2 months after hAECs transplantation. GFP positive staining co-localized with human anti-nuclear staining in ovarian stroma (Figure 4A-F) and in antral follicles (Figure 4G-L).

**Figure 5:**
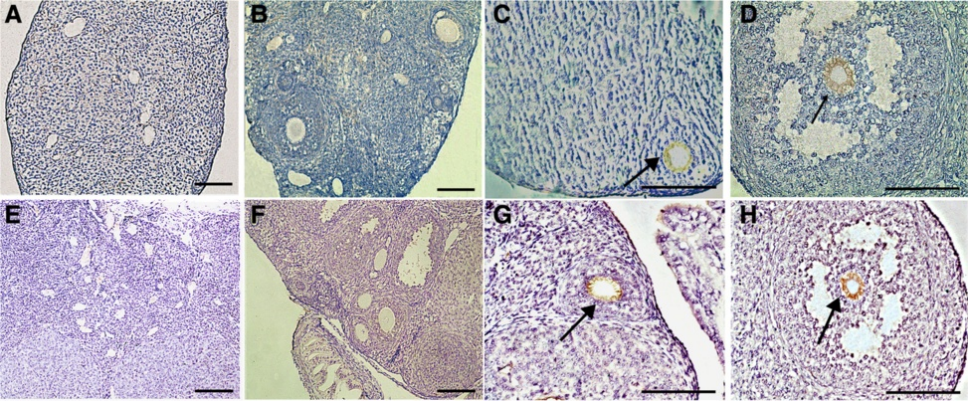
**Grafted cells detected by immunochemistry against human antigens. (A)** Human specific nuclear antigen expression was negative in recipient ovaries without hAECs transplantation. **(B)** Human nuclear antigen was not detected in some recipient ovaries two months after hAEC transplantation. **(C**, **D)** Human nuclear antigen was expressed in antral follicles in recipient ovaries two months after hAEC transplantation. **(E)** Human FSHR was not detected in recipient ovaries without hAEC transplantation. **(F)** Human FSHR was not detected in some recipient ovaries two months after hAEC transplantation. **(G**, **H)** Human FSHR were detected in recipient ovaries two months after hAEC transplantation. Arrows indicate human antigen expression in granulosa cells surrounding the ovum, whereas other granulosa cells were negative controls. Scale bars = 100 μm. hAECs, human amniotic epithelial cells.

Immunohistochemical analysis with human nuclear antigen was used to measure survival and differentiation of grafted hAECs. Human specific nuclei were expressed in some follicles of recipient ovaries two months after hAECs transplantation (Figure 5C, D), whereas human-specific nuclear expression was negative for ovaries that did not receive hAECs transplantation (Figure 5A). Human-specific nuclear expression was also detected in granulosa cells near the ovum, whereas the other granulosa cells were not stained (Figure 5C, D). Next, human follicle-stimulating hormone receptor (FSHR) was used to characterize the grafted cells. FSHR, a glycoprotein hormone receptor, can serve as a granulosa cell marker and is required for normal ovarian development and follicle maturation in females [21]. In addition, human FSHR can also be used as a human cell transplantation tracking marker. Using an immunochemical assay, human FSHR staining patterns were observed to be similar to human-specific nuclear antigen and human FSHR was only detected in cells proximal to the eggs in ovarian tissue two months after hAECs transplantation (Figure 5G, H).

These results strongly suggest that a portion of hAECs-derived cells were grafted to the chemotherapeutically murine ovarian tissue and participated in follicle production.

### Grafted hAECs partially restore ovarian function in chemotherapy-treated mice

**Figure 6:**
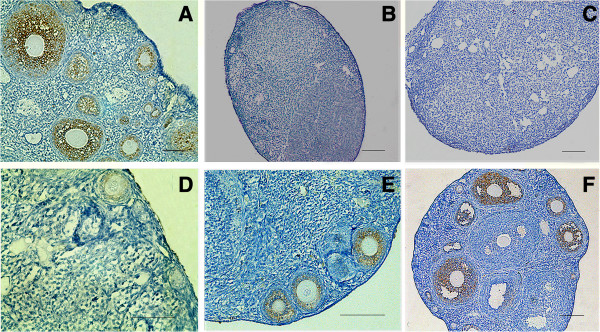
**AMH expression in recipient mouse ovaries. (A)** AMH was expressed in all granulosa cells of primary, preantral and small antral follicles in normal ovaries. **(B)** AMH expression disappeared in stromal and atretic follicles in recipients ovaries without hAECs transplantation two months after chemotherapy. **(C)** AMH expression was negative in recipient ovaries seven days after hAEC transplantation. However, AMH expressions were detected in recipient ovaries after hAEC transplantation for 14 days **(D)** and 21 days **(E)**. **(F)** AMH expression patterns are strong in recipient ovaries two months after hAEC transplantation; data are consistent with that from control normal ovaries. Scale bars = 100 μm. AMH, Anti-Müllerian hormone; hAEC, human amniotic epithelial cell.

AMH is a member of the transforming growth factor β family of growth and differentiation factors. In humans, it is encoded by the AMH gene which is expressed by ovarian granulosa cells in reproductive ages. AMH controls the formation of primary follicles by inhibiting excessive follicular recruitment by FSH. AMH is strongly correlated to the follicle pool size. It, therefore, has a role in folliculogenesis and can be used as a marker of ovarian aging, responsiveness and pathophysiology [22]. As shown in Figure 6A, AMH expression was strong in granulosa cells of preantral and small antral follicles in normal ovaries, but AMH was not expressed in ovaries sterilized with chemotherapy (Figure 6B). AMH was not detected in recipient ovaries seven days after hAECs transplantation (Figure 6C); however, AMH expression appeared in primary follicles of recipient ovaries 14 days after hAECs transplantation (Figure 6D), and strong expression of AMH was observed in recipient ovaries 21 days and 2 months after hAECs transplantation (Figure 6E, F).

These results demonstrate that ovarian function can be partially restored in sterile recipient females by transplantation of hAECs.

## Discussion

Infertility is functionally defined as the inability of a person to contribute to conception. Cancer treatment, such as surgery, chemotherapeutic or radiologic treatments, can decrease the number of primordial follicles, affect hormonal balance, or interfere with the functioning of the ovaries, fallopian tubes, uterus or cervix. Anatomic or vascular changes to the uterus, cervix or vagina from surgery or radiation may also prevent natural conception and successful pregnancy, requiring assisted reproductive technology or use of a gestational carrier. Unfortunately, fertility preservation methods are used infrequently for people with cancer, and the quality of life of cancer survivors after chemotherapeutic or radiologic treatments is an increasing concern, specifically with respect to restoring gonadal function and reviving fertility [23]. Stem cells are unique cells with the potential for not only self-renewal but also the ability to differentiate into specialized progeny. Evidence suggests that transplantation of stem cells from bone marrow can restore ovarian function that has been destroyed by gonadotoxic insults [8,10]. Recently, Ghadami and colleagues reported that bone marrow stem cell transplantation could restore follicular maturation and steroid hormone production in a follitropin receptor knockout (FORKO) mouse model used to study primary ovarian failure [24]. Irrespective of current stem cell controversies, general agreement exists regarding the utility of stem cell therapy, such as bone marrow stem cells, for improving ovarian function.

Adult bone marrow (BM) is the most common source of clinically used mesenchymal stem cells (MSCs). However, adult BM has limitations. First, the frequency of MSCs in adult BM is low. Second, harvesting BM from a patient is invasive. Therefore, alternative sources of MSCs are needed for future clinical applications. Compared with BM or other adult stem cells, hAECs have enormous potential for serving as a stem cell source for basic science and regenerative medicine. hAECs can differentiate into cells from all three embryonic tissue types, such as ectoderm cells (neural cells), mesodermal cells (cardiomyocytes) and endodermal cells (hepatocytes and pancreatic cells) [11-13]. Moreover, hAECs can be isolated from term placentas without the sacrifice of human embryos and the cells can be readily cryopreserved for cell banking. Therefore, hAECs represent a powerful tool for cellular therapy for different human diseases, such as neurodegenerative diseases and nervous system injuries [25], hepatic fibrosis [26] and myocardial infarction (cardiac and smooth muscles) [27]. However, at this time whether hAECs can restore ovarian function is unclear.

To address this, we analyzed hAECs for expression of germ cell markers. As expected from a previous report [11,12], we observed consistent expression of OCT4, NANOG and CD117 expression in hAECs. Then, we measured expression of additional germ-cell-specific genes of hAECs. In hAECs, we did not observe expression of genes known to be restricted in the expression of germ cell differentiation. Recent studies showed that hAECs can express germ-cell-specific markers [16], suggesting that hAECs can restore ovarian function after transplantation into chemotherapeutically damaged ovaries.

Using a preclinical mouse model of chemotherapy-induced ovarian failure, hAECs were transfected with GFP lenti-virus and were transplanted via tail vein injection one week after chemotherapy-induced sterility. Timing of transplantation was guided by the report of Lee and colleagues who suggested that the maximum benefit of stem cell transplantation was achieved when transplantation occurred one week after chemotherapy [8]. One week after transplantation, hAECs-derived GFP-positive cells were observed in ovaries, and primordial follicles could be found in recipient ovaries two weeks after hAECs transplantation. Various stages of developing follicles, including GFP-stained follicles could be observed three weeks to two months after hAECs transplantation. Double-staining with GFP and human specific nuclear antigen demonstrated that GFP-positive cells in recipient ovaries were derived from grafted hAECs. Importantly, human specific nuclear antigen expression was observed in granulosa cells surrounding the ovum, but not egg cells. Furthermore, human FSHR expression patterns in recipient ovarian follicles were localized to all ovarian granulosa cells of the Graafian follicles [20]. Human FSHR expression in sterilized ovaries indicated that a portion of the grafted hAECs differentiated into granulosa cells, but not germ cells.

Further, the ovarian function of infertile mice was improved after hAECs transplantation. Because AMH is correlated with the size of the follicle pool, it can be used as a marker of ovarian aging, responsiveness and pathophysiology [22,28], and serum AMH has been used as a clinical marker of ovarian function. AMH has been reported to be a promising marker for premature ovarian failure in both healthy women and those with Turner syndrome [29]. AMH measurements at cancer diagnosis also predict long-term ovarian function after chemotherapy; so this marker may better predict chemotherapy-related risk to future fertility [30]. Our results showed that AMH was strongly expressed in the ovary of infertile mice three weeks to two months after transplantation. However, AMH expression was not detected in recipient ovaries until two weeks after transplantation, suggesting that after transplantation at least two weeks must elapse before restoration of ovarian function is obtained in infertile mice.

The mechanisms of stem cell transplantation for the treatment of different disorders remain unclear at this time, and controversies exist about their use. Evidence suggests that the microenvironment of injury could affect the migration, adherence and differentiation of transplanted stem cells [31-33]. Transplanted stem cells might transdifferentiate to tissue-specific cells, or fuse with the existing native cells, to improve the organ function by contributing their own genetic and cellular materials [33,34]. The development of ovarian follicles requires complex cell-cell interactions, as well as communication between somatic and germ cells. In the developing oocyte, granulosa cells surround the oocyte, support its growth, and provide hormonal supplementation, so normal granulosa cell communication and development is critical for oocyte growth [35]. This is important because a previous study suggested that cyclophosphamide (CTX) metabolites might decrease granulosa cell function and induce ovarian toxicity during CTX therapy [36].

In this study, we found that GFP-labeled cells initially entered into ovarian stroma, and then distributed proximal to the follicles, and finally surrounded the oocytes (Figures 3 and 4). Further, human FSHR could be detected in cells surrounding the oocytes in ovarian tissue two months after transplantation. Thus, hAECs may integrate into the ovaries of infertile mice, and some hAECs may transdifferentiate to granulosa cells and direct follicle development. Recently, evidence was reported that ovaries of reproductive-age women, similar to adult mice, possess rare germ line stem cells [17,37], which might explain the observation that ovarian function after chemotherapy can be restored spontaneously in some cases [8]. However, our results revealed that all of the sterilized mouse ovaries produced follicles after hAEC transplantation, whereas only a few of control ovaries responded in this manner. Thus, we believe that hAECs function primarily by reactivating host gem line stem cells. Additional experiments are necessary to elucidate the exact mechanisms by which stem cell transplantation restores ovarian function.

Previous studies indicate that amniotic epithelial cells had no karyotypic abnormalities or transformation potential *in vitro* and no tumorigenic effects *in vivo*[14]. Evidence also suggested that the amniotic epithelial cells had immunosuppressive properties [38,39]. Here C57BL/6 mice (not SCID mice) were used to model ovarian failure. Mice receive no immunosuppressive agents before hAEC transplantation, and no transplant-related deaths were observed after hAEC transplantation. However, the immunological rejection of hAECs needs to be further elucidated before clinical application.

## Conclusion

In summary, hAECs derived from term placentas after live birth may have the intrinsic ability to restore folliculogenesis, and hAEC transplantation may be a testable clinical strategy for preserving or resurrecting ovarian function in female cancer patients after chemotherapy. Our findings have implications for the future use of stem cells for the improvement of the quality of life in cancer survivors.

## Abbreviations

AMH: Anti-Müllerian hormone; BLIMP1: B-lymphocyte-induced maturation protein; BM: Bone marrow; c-MOS: Oocyte maturation factor mos; CTX: Cyclophosphamide; DAZL: Deleted in azoospermia-like; DMSO: Dimethyl sulfoxide; EGFP: Green fluorescent protein; FSHR: Follicular stimulating hormone receptor; GFP: Green fluorescent protein; GnRH: Gonadotropin-releasing hormone; H&E: Hemotoxylin and eosin; hAECs: Human amniotic epithelial cells; MSCs: Mesenchymal stem cells; PBS: Phosphate-buffered saline; PGCs: Primordial germ cells; SCID: Severe combined immunodeficiency; SCP1 and SCP3: Syntaptonemal complex protein 1 and 3; STELLA: Developmental pluripotency-associated protein 3, stella-related protein; STRA8: Stimulated by retinoic acid gene 8; VASA: Probable ATP-dependent RNA helicase DDX4, vasa homolog.

## Competing interests

The authors have no financial or non-financial competing interests.

## Authors’ contributions

FW and XY carried out the animal model, surgery and sample collection. LW carried out the molecular analysis. FW performed the pathophysiological examination. FW, DL and LG participated in data acquisition and performed statistical analysis. FW and DL participated in data analysis and manuscript editing. DL designed, conceived of the study, and drafted the manuscript. All authors read and approved the final manuscript.

## Supplementary Material

Additional file 1: Table S1 Real-time PCR primer sequences. **Table S2.** Real-time PCR in human amniotic epithelial cells (hAECs). Mean normalized expression of genes in hAECs were calculated by 2(-ΔCt). Results are shown as mean and standard deviation of three experiments. 18 s RNA was used as internal control. Click here for file
