# Expression of Concern: Human amniotic epithelial cells can differentiate into granulosa cells and restore folliculogenesis in a mouse model of chemotherapy-induced premature ovarian failure

**DOI:** 10.1186/s13287-015-0234-7

**Published:** 2015-12-08

**Authors:** Rocky S. Tuan, Timothy O’Brien

**Affiliations:** 1 Center for Cellular and Molecular Engineering, Department of Orthopaedic Surgery, University of Pittsburgh School of Medicine, Pittsburgh, 15219 PA USA; 2 REMEDI, CURAM, National University of Ireland Galway, Galway, Ireland

## Expression of concern

After publication of this article [1] it was brought to the Editors’ attention that it was previously published by the *OMICS Journal of Stem Cell Research & Therapy*. The authors report that they requested retraction before submission to *Stem Cell Research & Therapy* but did not receive a response*.* The *OMICS Journal of Stem Cell Research & Therapy* has now removed the article from its website without a formal retraction notice. Given that this article is no longer a duplicate publication, we have decided not to retract this version. We are issuing this Expression of Concern to alert readers should they find versions of the *OMICS Journal of Stem Cell Research & Therapy* article before it was removed from that journal’s website. We apologize to all affected parties.
