# Supplementary material for: Human amniotic epithelial cells can differentiate into granulosa cells and restore folliculogenesis in a mouse model of chemotherapy-induced premature ovarian failure
Source: Stem Cell Res Ther. 2013 Oct 14;4(5):124. doi: 10.1186/scrt335 (PMC3854701; doi:10.1186/scrt335)
Supplement: Additional file 1: Table S1 — Real-time PCR primer sequences. Table S2. Real-time PCR in human amniotic epithelial cells (hAECs). Mean normalized expression of genes in hAECs were calculated by 2(-ΔCt). Results are shown as mean and standard deviation of three experiments. 18 s RNA was used as internal control. [file scrt335-S1.doc]

Additional file 1: Table S1

| Gene product | Forward(F) and reverse(R) primers(5’ → 3’) | Size(bp) |
| --- | --- | --- |
| *NANOG* | F: GGGCCTGAAGAAAACTATCCATCC  R: TGCTATTCTTCGGCCAGTTGTTTT | 400 |
| *OCT4* | F: GGCCCGAAAGAGAAAGCGAACC  R: ACCCAGCAGCCTCAAAATCCTCTC | 224 |
| *CD117* | F:CCACACCCTGTTCACTCCTT  R:TTCTGGGAAACTCCCATTTG | 206 |
| *HLA-DR* | F: CAGTTCCTCGGAGTGGAGAG  R: CTCAGCATCTTGCTCTGTGC | 115 |
| *BLIMP1* | F: AAGTGTAACTCCAGCACTGTG  R: CCAAAACGTGTGCCCTTTGGTATG | 290 |
| *STELLA* | F: CTCAAATCTCCTCCGAGACG  R: TTCGATTTCCCTGAGGACTG | 137 |
| *DAZL* | F: AATGACGTGGATGTGCAGAA  R: AACTGTGGTGGAGGAGGATG | 152 |
| *VASA* | F: TTGGGAAGCAGAAATCAACC  R: AAACCACCCATTGTGGATGT | 240 |
| *c-MOS* | F: CGGTGTTCCTGTGGCCATAA  R: AGCAGGCCGTTCACAACATC | 330 |
| *STRA8* | F:TCGTCTCCGCGGCCATCTCC | 153 |
|  | R: TGTCCTTCACGCTGCCCTCG |  |
| *SCP1* | F: CAAAAGCCCTTTGCATTGTT | 225 |
|  | R: CTCAAACACGGGCAAGAAAT |  |
| *SCP3* | F: TATGGTGTCCTCCGGAAAAA | 238 |
|  | R: AACTCCAACTCCTTCCAGCA |  |
| *18s RNA* | F: CGTTGATTAAGTCCCTGCCCTT  R: TCAAGTTCGACCGTCTTCTCAG | 202 |

Additional file 1: Table S2

|  | hAEC-1(10^-7^) | hAEC-2(10^-7^) | hAEC-3(10^-7^) | hAEC-4(10^-7^) | hAEC-5(10^-7^) | hAEC-6(10^-7^) |
| --- | --- | --- | --- | --- | --- | --- |
| *NANOG* | 92.3±23.21 | 75.3 ± 22.42 | 54.2±3.73 | 81.1±11.72 | 68.0±11.92 | 67.6±14.13 |
| *OCT4* | 109±12.81 | 132±17.90 | 81.8±10.40 | 76.8±12.91 | 141±13.21 | 128±27.20 |
| *CD117* | 112±9.14 | 229±37.52 | 187±21.81 | 281±34.70 | 834±8.69 | 676±26.40 |
| *HLA-DR* | 4.98±0.09 | 12.9±1.53 | 7.60±0.850 | 13.4±10.2 | 43.1±0 | 12.1±5.39 |
| *BLIMP1* | 44.8±10.3 | 32.5±8.37 | 32.9±.883 | 27.9±2.49 | 43.6±8.66 | 36.0±2.25 |
| *STELLA* | 4.72±2.64 | 4.90±2.47 | 4.41±2.55 | 6.48±0 | 5.83±2.24 | 3.92±2.76 |
| *DAZL* | 2.34±0.85 | 1.51±1.16 | 1.78±0.40 | 4.70±2.68 | 2.44±1.15 | 4.38±0.56 |
| *VASA* | 1.14±0.398 | 1.65±0.721 | 1.28±0.898 | 1.91±0.124 | 1.56±0 | 1.02±0 |
| *STRA8* | 35.9±7.52 | 34.7±6.40 | 35.1±5.52 | 44.4±8.26 | 39.8±7.80 | 34.6±8.66 |
| *c-MOS* | 11.6±8.23 | 8.96±7.86 | 9.55±1.54 | 10.4±5.41 | 12.2±2.27 | 13.2±1.22 |
| *SCP1* | 6.29±0 | 4.16±1.36 | 3.42±0 | 7.3±2.83 | 7.33±5.33 | 3.88±2.82 |
| *SCP3* | 8.22±2.12 | 6.82±3.82 | 7.73±6.87 | 7.60±2.68 | 8.65±5.96 | 9.45±7.15 |
